# Associations Between Fear of COVID-19, Affective Symptoms and Risk Perception Among Community-Dwelling Older Adults During a COVID-19 Lockdown

**DOI:** 10.3389/fpsyg.2021.638831

**Published:** 2021-03-23

**Authors:** Madeline F. Y. Han, Rathi Mahendran, Junhong Yu

**Affiliations:** ^1^Department of Psychological Medicine, Yong Loo Lin School of Medicine, National University of Singapore, Singapore, Singapore; ^2^Academic Development Department, Duke-NUS Medical School, Singapore, Singapore

**Keywords:** COVID-19, fear, depression, anxiety, risk perception, older adults

## Abstract

Fear is a common and potentially distressful psychological response to the current COVID-19 pandemic. The factors associated with such fear remains relatively unstudied among older adults. We investigated if fear of COVID-19 could be associated with a combination of psychological factors such as anxiety and depressive symptoms, and risk perception of COVID-19, and demographic factors in a community sample of older adults. Older adults (*N* = 413, *M*_age_ = 69.09 years, *SD* = 5.45) completed measures of fear of COVID-19, anxiety and depressive symptoms, and risk perception of COVID-19, during a COVID-19 lockdown. These variables, together with demographics, were fitted to a structural equation model. Anxiety and depressive symptoms were highly correlated with each other and were combined into the higher order latent variable of affective symptoms for analyses. The final model revealed that fear of COVID-19 was positively associated with psychological factors of affective symptoms and risk perception. Older age was associated with greater fear of COVID-19. Our findings showed that fear of COVID-19 can be a projection of pre-existing affective symptoms and inflated risk perceptions and highlighted the need to address the incorrect risk perceptions of COVID-19 and socio-affective issues among older adults in the community.

## Introduction

The novel coronavirus disease (COVID-19) was first discovered in Wuhan, China in December 2019, and has since been declared a global pandemic by the World Health Organization in March 2020 (World Health Organization, 2020b). In response to the health crisis, countries worldwide have implemented a series of public health and social distancing measures to curb the spread of COVID-19.

Singapore saw its first confirmed COVID-19 case on January 23, 2020, and the emergence of its first cluster of community cases on February 04, 2020 (Goh, 2020). As the threat of COVID-19 grew, the government raised its Disease Outbreak Response System Condition^1^ level from yellow to orange on February 7, which signaled to the public that COVID-19 is severe and spreads easily, with moderate disruptions to daily life (Khalik, 2020). Shortly after, panic buying was observed in the community, with household items such as toilet paper and instant noodles being wiped out in supermarkets (Baker, 2020).

The COVID-19 situation began to worsen, and Singapore reported the first two local COVID-19 deaths on March 21. Eventually, on April 3, a “circuit breaker” measure (gov.sg, 2020a; Ministry of Health, 2020) was put in place to prevent further spread of the disease. The circuit breaker was implemented from April 07 to June 01, 2020, which comprised a series of measures such as a nationwide partial lockdown and closure of non-essential workplaces. Wearing of masks was mandatory and gathering in groups was banned. During this period, confirmed cases of COVID-19 in local communities still continued to rise substantially, and new clusters started to form and spread rapidly among foreign workers’ dormitories. On April 20, Singapore reached its peak of 1,426 new COVID-19 cases, with the majority of them coming from dormitories (Baker, 2020).

Following the circuit breaker, a “Phase 1” measure was implemented from June 02 to 18, 2020 (gov.sg, 2020b). Phase 1 was part of a three-phased approach aimed to progressively lift the circuit breaker measures, to ensure that transmission rates of COVID-19 remain under control (gov.sg, 2020b). While there was gradual re-opening of some businesses and activities, many restrictions in Phase 1 were largely similar to those in the circuit breaker.

With the backdrop of high COVID-19 cases and the constantly evolving situation locally and globally, it is imperative to examine the psychological and mental health impacts COVID-19 brings to individuals. One psychological response commonly reported is fear toward COVID-19 (Ahorsu et al., 2020a; Pakpour and Griffiths, 2020; Schimmenti et al., 2020). Fear is an adaptive emotion fundamental for survival, which serves to prepare the individual for behavioral responses to potential threats (Garcia, 2017). Fear may occur in response to specific stimuli in the present environment, or in anticipation of future or imagined events that pose a threat to oneself.

In the context of the COVID-19 pandemic, fear may be beneficial as it motivates preventive behavior such as hand-washing and social distancing (Harper et al., 2020). However, fear can become maladaptive when it is excessive, leading to significant levels of distress and irrational behaviors at both the individual and population level. For the former, fear of COVID-19 may exacerbate pre-existing mental health conditions (Ho et al., 2020), while in several cases, fear may lead to suicidal behavior (Dsouza et al., 2020; Mamun and Griffiths, 2020; Sher, 2020). For the latter, fear predicts panic buying (Arafat et al., 2020) as well as racist and discriminatory responses (Devakumar et al., 2020). As such, it is important to investigate the variables associated with fear of COVID-19, in order to ensure fear is well-managed and that detrimental consequences resulting from excessive fear is minimized.

Fear of COVID-19 is associated with some major psychological factors, according to the existing literature. First, affective symptoms such as those of depression and anxiety may be associated with fear. Depression refers to a state of low mood characterized by general negative views of the self, world and the future (Beck, 1979), while anxiety is an emotion characterized by internal feelings of tension and worry, which may be accompanied with physical responses such as sweating and increased heart rate (American Psychological Association, 2021). Symptoms of depression and anxiety are common psychological reactions to COVID-19 (Rajkumar, 2020), and correlational evidence thus far suggests that fear of COVID-19 is positively associated with depression and anxiety (Harper et al., 2020; Satici et al., 2020). Further, another study found that depression and anxiety, amongst other variables, play a mediating role between fear of COVID-19 and positivity (Bakioğlu et al., 2020). Given an unprecedented time with high infection and transmission rates of COVID-19, the co-occurrence and association between fear, anxiety and depression are not surprising. Hence, based on the aforementioned evidence in the literature, we would expect fear of COVID-19 to be associated with both depressive and anxiety symptoms.

Another related psychological factor may be risk perception. Risk perception is a subjective judgment an individual makes on the likelihood of negative occurrences (Paek and Hove, 2017), and it is often influenced by cognitive, emotional, social, and cultural factors (Douglas and Wildavsky, 1983; Slovic, 2000; Loewenstein et al., 2001; Van der Linden, 2015). While literature on risk perception from previous infectious diseases remains scant (De Zwart et al., 2007), many laboratory studies suggest an interplay between emotions and heightened risk perception, with fearful individuals exhibiting increased risk perception of negative outcomes following fear-relevant stimuli. This association has been found in various groups of individuals, such as those with fears of snakes and spiders (Tomarken et al., 1989; Amin and Lovibond, 1997; Kennedy et al., 1997), fear of contamination (Olatunji et al., 2006), and socially anxious individuals (de Jong et al., 1998). Additionally, a field experiment on the terrorist attack on September 11, 2001 has shown that fearful individuals had higher risk estimates and more plans for precautionary actions (Lerner et al., 2003).

While studies have shown known associations between risk perception and fear, it is important to note that they are of conceptually different constructs. Inflated risk perceptions may not always be accompanied by increased fear levels. For example, repeated prolonged exposure to the fear stimulus may ultimately reduce or eliminate fear entirely, but judgment of risks may still remain the same as before (as risk can be influenced by factors other than emotional ones). Nonetheless, in the context of the COVID-19 pandemic where there is no previous prolonged exposure, we would expect risk perception and fear of COVID-19 to be positively related. Individuals with increased risk perception may also have higher fear levels of COVID-19.

Given these previous fear-related findings, we put together a model of fear of COVID-19 which explores the relationships with anxiety and depressive symptoms, and risk perception of COVID-19. We tested this model using structural equation modeling on cross-sectional data collected from community-dwelling older adults during a COVID-19 lockdown in Singapore. A secondary aim of our study was to investigate potential demographic factors associated with fear of COVID-19, which would be useful to identify potential demographical subgroups who are more fearful of COVID-19.

## Materials and Methods

### Participants and Procedures

Participants in our study were recruited from the Community Health and Intergenerational study, an existing cohort study involving community-dwelling older adults in Singapore (Lee R. Z. Y. et al., 2020). These participants were recruited via door-to-door recruitment within various housing estates in the Western region of Singapore. Participants from the Community Health and Intergenerational study were invited to join the current study based on the following inclusion criteria: (a) age between 60 and 99 years, (b) literate in either English or Mandarin, and (c) no diagnosis of Dementia. These participants must also have indicated their consent to be re-contacted for future studies, and to donate their coded data for future research in the Community Health and Intergenerational study’s consent form. A total of 582 older adults fulfilled the inclusion criteria and were contacted over the phone for their interest in the current study.

Participation to the current study was voluntary, and consent (verbal or written) was obtained from all participants before enrolment. Of the 582 contacted, 454 older adults provided consent to participate. However, 40 failed to submit responses, resulting in a response rate of 91.2%. 1 had missing data and were excluded from further analysis. This resulted in a final sample of 413 participants. The socio-demographic characteristics of the sample are illustrated in Table 1.

**TABLE 1:** Socio-demographic characteristics of the sample (*N* = 413).

Socio-demographic characteristics	*M (SD)* or *n* (%)
Age (years)	69.09 (5.45)
**Gender**	
Female	270 (65.4)
Male	143 (34.6)
Years of education	13.58 (3.83)
**Marital status**	
Single	48 (11.6)
Married	312 (75.5)
Widowed	33 (8.0)
Divorced or separated	19 (4.6)
Undisclosed	1 (0.2)
**Housing type**	
1–2 room public housing	16 (3.9)
3 room public housing	24 (5.8)
4–5 room public housing	128 (31.0)
Executive or maisonette	52 (12.6)
Private apartment or condominium	95 (23.0)
Landed housing	96 (23.2)
Undisclosed	2 (0.5)

**M*, mean; *SD*, standard deviation.*

Participants completed the study questionnaires in either English or Mandarin, on an online survey platform (Qualtrics) or through mail. Participants who completed all questionnaires were remunerated with $10. The study received ethics approval by the National University of Singapore Institutional Review Board (S-20-118E). Data collection took place from May 11 to June 05, 2020.

### Measures

#### COVID-19 Fear Inventory

The COVID-19 Fear Inventory is a newly constructed 13-item scale developed by the authors to assess fear of COVID-19 and its associated concerns. The inventory was adapted from the Ebola Fear Inventory (Blakey et al., 2015) and the Swine Flu Anxiety Items (Wheaton et al., 2012), which were used to assess Ebola and H1N1 (swine flu) fears respectively. Participants rated their agreement with each item on a 5-point Likert scale ranging from 1 (“not at all”) to 5 (“very much”). Higher scores corresponded to higher fear levels of COVID-19. The full list of items in the COVID-19 Fear Inventory is presented in Supplementary Table 1.

#### 15-Item Geriatric Depression Scale (GDS-15)

The GDS-15 (Yesavage and Sheikh, 1986) is a shortened version of the Geriatric Depression Scale used to assess depressive symptoms among older adults. Participants responded “yes” or “no” to 15 items, with 1 point awarded to each response indicative of depressive symptoms. Higher scores corresponded to higher levels of depressive symptoms. The scale demonstrated good psychometric validity in the local context (Nyunt et al., 2009), achieving the optimal cut-off of 4/5 used in most studies (Wancata et al., 2006).

#### Geriatric Anxiety Inventory—Short Form (GAI-SF)

The GAI-SF (Byrne and Pachana, 2011) is a shortened version of the Geriatric Anxiety Inventory (GAI), which consists of 5 items that measures anxiety symptoms among older adults. Participants were asked to indicate “agree” or “disagree” for each item, with higher scores suggesting higher levels of anxiety symptoms. The GAI-SF was found to have good psychometric properties for assessing anxiety symptoms and may be used as a useful alternative to the original GAI (Johnco et al., 2015).

#### Risk of Infection Questionnaire

The Risk of Infection Questionnaire is a 10-item scale constructed by the authors which aimed to assess perceived risk of COVID-19 infection. Participants rated the likelihood of being infected with COVID-19 under different situations (e.g., in a train) or from contact with commonplace objects (e.g., lift buttons). Each item was rated on a 4-point Likert scale ranging from 1 (“very unlikely”) to 4 (“very likely”). Higher scores indicated heightened risk perception of COVID-19 infection. The full list of items in the questionnaire can be found in Supplementary Table 2. At the point of study conceptualization, there were no similar scales available for the purpose of measuring perceived risk of COVID-19 infection.

### Data Analysis Strategy

First, we conducted a confirmatory factor analysis (CFA) to establish the factor structure of the COVID-19 Fear Inventory and Risk of Infection Questionnaire. As both scales were newly developed, it was important to ensure that the included items for analysis fit a hypothesized measurement model as well as to partial out measurement error. Individual items from each scale were fitted into latent variables (i.e., fear and risk perception latent variable), which were then used for further analysis. Likewise, latent variables for depressive and anxiety symptoms were constructed from items in GDS and GAI-SF, respectively, to partial out their measurement error.

Next, we used structural equation modeling (SEM) to model the relationship between fear, anxiety, and depressive symptoms, and risk perception, with age, gender, and years of education included as demographic covariates. The latent variables for depressive and anxiety symptoms were loaded on to a higher-level latent variable—affective symptoms, which was used to predict fear, instead of having anxiety and depressive symptoms predict fear individually. Using SEM for our data analysis strategy was appropriate as the SEM approach is versatile enough for us to concurrently fit items into a measurement model, and to explore the relationships between the fitted latent variables.

For both CFA and SEM, robust maximum likelihood was used for parameters estimation, with analyses being carried out with the R package lavaan (Rosseel, 2012). The Root Mean Square Error of Approximation (RMSEA), Comparative Fit Index (CFI), Standardized Root Mean square Residual (SRMR), Akaike Information Criterion (AIC) and Bayesian Information Criterion (BIC) were used to assess model fit. CFI values > 0.90 were considered an acceptable fit (Hair et al., 2010), while SRMR and RMSEA values < 0.08 were indicative of an acceptable model (Hu and Bentler, 1999). Lower information criterion values correspond to better fit. The χ^2^ test of difference was used to compare the fit between models.

Correlations between fear and other continuous variables were examined using Pearson correlation coefficients. Statistical significance was set at *p* < 0.05. A *post hoc* power analysis on the R package SemPower (Moshagen and Erdfelder, 2016) was also conducted to determine the achieved power of the final SEM model. All analyses were performed in R 4.0.0. The R code for executing these analyses is available at https://osf.io/byfc4.

## Results

### Factor Analyses of COVID-19 Fear Inventory and Risk of Infection Questionnaire

As COVID-19 Fear Inventory is a newly developed scale, we first employed CFA to evaluate its factor structure. Items 7, 8, 10 and 11 were excluded from analysis due to low factor loadings and other theoretical considerations. For instance, items 7 and 8 relate to the threat of COVID-19 influencing one to practice social distancing and use protective supplies. As these actions were made compulsory by the government (Ang and Phua, 2020; The Straits Times, 2020), it may be possible that individuals were motivated to follow these measures as a result of fear and obedience to the rules, not by fear alone. For item 10 [“To what extent do you engage in panic buying (i.e., buying large amounts of protective supplies and other essential items because of the threat of COVID-19)?”], panic buying only occurred during the early stages of the pandemic, and it was not prevalent during the period of data collection. Item 11 relates to the fear of one’s household income being affected due to the threat of COVID-19. As household income was supplemented with government financial packages (gov.sg, 2020c), this item would not be very relevant. Taken together, these items may not be true measures of fear, and were removed from the model. This resulted in a 9-item scale with unstandardized factor loadings ranging from 0.55 to 1.0 (*p* < 0.001). The results also indicated that a single factor best fits the model. Supplementary Table 3 shows the fit indices of the tested CFA models.

Similarly, CFA was conducted to validate the factor structure of the Risk of Infection Questionnaire. The results showed that items 2 (“pressing the lift buttons”) and 9 (“being around doctors, nurses and other hospital staff”) had relatively lower factor loadings. Furthermore, situations described in items 2 and 9 have some overlaps with those in items 3 and 10 (i.e., items 2 and 3 both depict a lift setting, while items 9 and 10 both depict a hospital setting). Because of the above considerations, items 2 and 9 were excluded from subsequent analysis, thus resulting in a final 8-item scale with unstandardized factor loadings ranging from 1.0 to 1.3 (*p* < 0.001). The results indicated that a single factor best fits the model. Fit indices of the tested CFA models can be found in Supplementary Table 3.

### Descriptive Statistics of Study Measures and Correlation Analysis of Continuous Variables

Table 2 presents the mean scores, standard deviations, and Cronbach’s alpha for the total scores of the study measures included for analysis in the SEM model. Pearson correlations were conducted to examine the relationships among them. Table 3 presents the results obtained. As shown, there were significant positive correlations between fear and age, depressive symptoms, anxiety symptoms, and risk perception; no significant correlations were found between fear and years of education.

**TABLE 2:** Descriptive statistics of key study measure.

Measure	*M (SD)*	Cronbach’s α
COVID-19 Fear Inventory	28.66 (8.45)	0.90
15-item Geriatric Depression Scale	1.70 (2.24)	0.80
Geriatric Anxiety Inventory—Short Form	0.47 (1.06)	0.70
Risk of Infection Questionnaire	24.80 (3.87)	0.84

*M, mean; SD, standard deviation.*

**TABLE 3:** Pearson’s correlation coefficients between fear and the continuous predictors.

Variable	1	2	3	4	5	6
(1) Fear	–					
(2) Depressive symptoms	0.29**	–				
(3) Anxiety symptoms	0.33**	0.80**	–			
(4) Risk perception	0.15**	0.01	0.03	–		
(5) Age	0.21**	0.00	–0.08	0.03	–	
(6) Years of education	–0.05	−0.11*	–0.07	–0.02	0.02	–

*p < 0.05, **p < 0.01.

### Findings From SEM

#### Baseline Models

**FIGURE 1:**
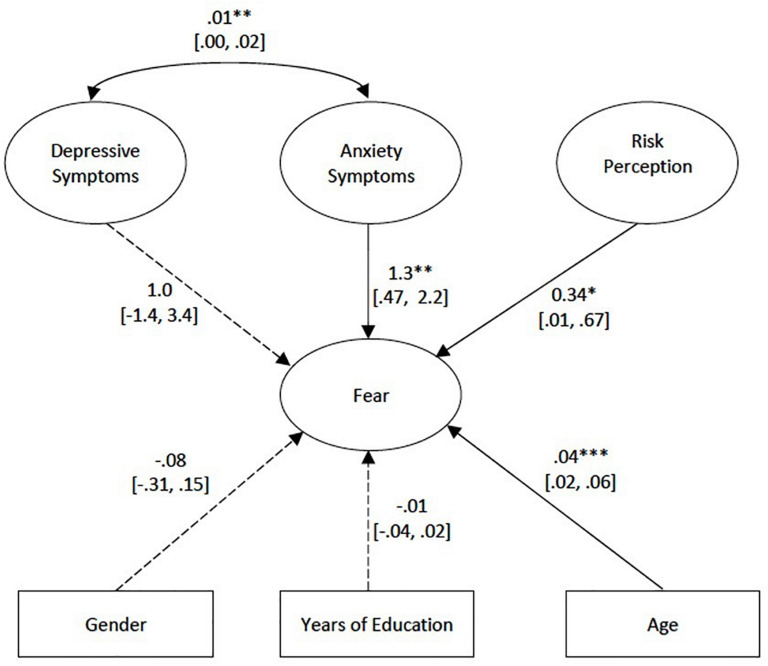
Baseline model. Unstandardized regression estimates are shown. Solid lines are significant at *p* < 0.05, while dotted lines are non-significant. Ovals represent latent variables, while rectangles represent observed variables. Individual indicators in the questionnaires are omitted in the figures for simplicity. Figures in brackets represent the Confidence Intervals. *p < 0.05, **p < 0.01, ***p < 0.001.

The latent variables and covariates were fitted into a baseline model which is illustrated in Figure 1. Our findings revealed that age was significantly associated with fear. As expected, anxiety and depressive symptoms were seen to be highly associated with each other (*p* < 0.01). Model fit indices of the model was unsatisfactory [χ^2^(693) = 1259.30, *p* < 0.001; RMSEA = 0.05; CFI = 0.86; SRMR = 0.06].

A second model was constructed to improve the model fit. In this model, only variables that were significantly associated with fear (i.e., age, affective symptoms of anxiety and depression, and risk perception) were included. Evaluation of model fit indices for the second model revealed that it did not have a reasonable fit [χ^2^(624) = 1100.13, *p* < 0.001; RMSEA = 0.05; CFI = 0.87; SRMR = 0.06].

#### Final Model

Modification indices were examined to determine whether the model fit could be improved. This resulted in a final model, which included three correlated errors from the COVID-19 Fear Inventory based on statistical and theoretical considerations. The first pair of error terms was items 1 (“To what extent are you afraid of being infected with COVID-19?”) and 2 (“If you are infected with COVID-19, to what extent are you afraid that you will be severely ill, or die?”). Correlated errors were expected as both items assessed fears of COVID-19 infecting the self. Similarly, there were overlaps between items 3 (“To what extent are you afraid that your loved ones will be infected with COVID-19?”) and 4 (“If your loved ones are infected with COVID-19, to what extent are you afraid that they will be severely ill, or die?”) as both items assessed fears of COVID-19 infecting loved ones. The last pair of error terms was items 12 (“To what extent are you worried that the threat of COVID-19 could affect your personal relationships?”) and 13 [“To what extent has the threat of COVID-19 influenced you to experience more negative emotions than usual (e.g., fear, worry, panic, etc.)?”]. Both items related to socio-emotional fears arising from COVID-19—strains on personal relationships may also affect one’s emotions in a negative manner.

**FIGURE 2:**
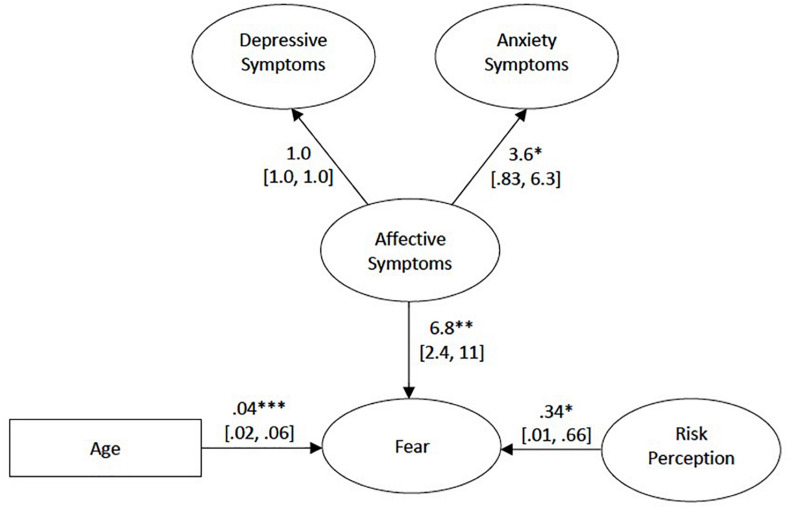
Final model. Unstandardized regression estimates are shown. Ovals represent latent variables, while the rectangle represents the observed variable. Correlated errors among the items in the COVID-19 Fear Inventory are not shown for clarity. Statistical significance for the path between depressive and affective symptoms was not tested as it was set to 1. Figures in brackets represent the Confidence Intervals. *p < 0.05, **p < 0.01, ***p < 0.001.

With the inclusion of the correlated errors, the model resulted in satisfactory fit indices [χ^2^(621) = 982.01, *p* < 0.001; RMSEA = 0.04; CFI = 0.91; SRMR = 0.06]. Furthermore, the χ^2^ test of difference between the models with and without the correlated errors was significant (Δχ^2^ = 72.94, Δdf = 3, *p* < 0.001), thus suggesting that the inclusion of the correlated errors significantly improved the model fit. Our *post hoc* power analyses on the model indicated that the sample size of *N* = 413 was associated with a power larger than >99.99% to reject a wrong model (with df = 621) with an amount of misspecification corresponding to RMSEA = 0.04 on alpha = 0.05. The results of the final model are presented in Figure 2.

## Discussion

The current study examined the association between psychological factors and fear of COVID-19 among community-dwelling older adults during a COVID-19 lockdown in Singapore. We also investigated potential demographic factors which could be related to fear of COVID-19. Our results indicated that affective symptoms (which include both depressive and anxiety symptoms), risk perception, and old age were associated with heightened fear of COVID-19.

Our findings revealed a strong interrelation between fear of COVID-19 and affective symptoms, suggesting the significant effect COVID-19 has on psychological well-being and mental health. While the relationship between fear and psychological distress is explored and well-supported in various studies (Bakioğlu et al., 2020; Harper et al., 2020; Satici et al., 2020), few studies (Ahorsu et al., 2020b) have explored this relationship specifically on the older adult population. Given that older adults are a high-risk group due to their increased physical and mental health vulnerabilities (Lee K. et al., 2020), more effort and attention should be given to explore COVID-19-related psychological distress among older adults.

The relationship between fear of COVID-19 and affective symptoms could be attributed to catastrophizing (Beck, 1979), a cognitive distortion commonly associated with anxiety and depression. Catastrophizing is a negative thinking style where one expects the worst possible outcome in a given situation. In the context of the COVID-19 pandemic, the negative outlook on reality could be accompanied with negative moods and emotions, including the experience of heightened fear levels. Speculatively, the association among the three variables seems to suggest the presence of a negative reinforcing loop; increased negativity may lead to higher fear levels toward COVID-19, which may in turn contribute to higher levels of depressive and anxiety symptoms (Bakioğlu et al., 2020; Satici et al., 2020). Given the cross-sectional nature of the current study, this hypothesis needs to be tested with future longitudinal datasets.

Our results indicated that higher fear levels of COVID-19 were associated with higher risk perceptions. Though the association has not been explored extensively in past epidemics, our finding was consistent with a previous study which investigated this association during the Ebola outbreak (Yang and Chu, 2018). Various theories in the existing literature have consistently pointed to the strong influence of emotions on risk assessment and decision-making. According to the appraisal-tendency framework, emotions influence one’s judgment and decision-making due to the different appraisals and cognitive responses accompanying emotions (Lerner and Keltner, 2000). Similarly, the risk-as-feelings hypothesis highlights the dominance of emotions in decision-making (Loewenstein et al., 2001). Both emotional reactions and cognitive assessments determine risk assessment; however, when conflict between the two arises, emotional reactions often override cognitive assessments and drive the eventual risk assessment and decision-making (Loewenstein et al., 2001). Based on the aforementioned theories, fearful individuals would perceive the COVID-19 pandemic as a high-risk event due to appraisals of lack of individual control, uncertainty, and unpleasantness (Smith and Ellsworth, 1985; Lazarus, 1991). In addition, the intense, immediate response and high mental imagery of fear (Loewenstein et al., 2001) would outweigh the cognitive evaluation of the actual threat posed by COVID-19. This suggests that fearful individuals are more likely to make inflated risk perceptions which are unrepresentative of actual risk levels.

As can be seen from our findings, older age was associated with greater fear of COVID-19. This could be attributed to the increased vulnerability to COVID-19 among the oldest-old. Clinical evidence thus far suggests that both COVID-19 mortality and fatality rates are associated with old age (Leung, 2020; Verity et al., 2020; Wang et al., 2020), with risks being especially high for the oldest-old. For example, studies in China and Italy reported that the case-fatality rate (CFR) of those 80 years and above was the highest at 14.8–20.2% (Novel Coronavirus Pneumonia Emergency Response Epidemiology, 2020; Onder et al., 2020). This was significantly higher than the CFR reported at 3.5–3.6% for older adults aged 60–69 years, and 8.0–12.8% for those aged 70 to 79 years (Novel Coronavirus Pneumonia Emergency Response Epidemiology, 2020; Onder et al., 2020). Considering the higher health risks posed to the oldest-old, it is not surprising to find that higher fear levels were found in this subgroup of older adults.

### Implications

The findings have several implications. First, they highlighted the characteristics of individuals who are more likely to react fearfully toward COVID-19, such as those with more severe affective symptoms (anxiety and depressive) and inflated risk perception, as well as those who are older. It is imperative that intervention programs take these factors into account, to help individuals alleviate and manage their fears.

Second, the present study highlights the concerns surrounding heightened risk perception of COVID-19. While existing research has demonstrated risk perception as an important tool in promoting protective and preventive behaviors during pandemics (e.g., hand-washing, wearing a surgical mask, and social distancing), incorrect and exaggerated perceptions of risk may potentially hinder the adoption of such behaviors (Dryhurst et al., 2020). Furthermore, heighted risk perceptions may lead to other serious and undesirable outcomes such as hoarding of essential health (e.g., medications) and protective supplies (e.g., personal protective equipment), resulting in a shortage of such supplies (Abrams and Greenhawt, 2020). Steps should be taken to ensure that risk perceptions are accurate and proportionate to the actual risk levels involved in COVID-19. Some appropriate risk communication methods include maintaining various platforms for active engagement between the public and healthcare professionals, and addressing common misunderstandings and misinformation through various means like public education, health hotlines, healthcare workers, and the community (World Health Organization, 2020a). Appropriate guidance is needed to prevent inflated risk perceptions of COVID-19 among individuals.

Third, our findings point to the need to address and alleviate the negative mental health consequences associated with fear of COVID-19. The affective symptoms of anxiety and depression should not be overlooked; if prolonged, these mental health consequences may result in longer-term problems than the pandemic itself (Ornell et al., 2020). In the current climate where offline, face-to-face contact is discouraged, it is important to consider other psychological interventions that allow individuals to continue to meet their mental health needs. Strategies include provision of online counseling services, health education programs (Liu et al., 2020), and telemental health services (Zhou et al., 2020).

### Limitations

The present study has a few limitations. First, due to the cross-sectional design of our study, we are unable to make inferences on the directionality of the relationships between studied variables. Second, as data were collected through self-reports, it may be possible that the data may be influenced by social desirability bias. Third, the lockdown conditions enforced during the circuit breaker may be unique to Singapore and may not be generalizable or comparable to those in other countries. While a complete lockdown was implemented in other countries, where individuals were not allowed to head out entirely, a partial lockdown was enforced in Singapore. Individuals could still head out for essential activities.

### Conclusion

In conclusion, our results show that fear of COVID-19 was positively associated with the affective symptoms of anxiety and depression, risk perception, and old age. Care needs to be given to these factors when designing intervention programs aimed to manage fears of COVID-19 among individuals. The results also highlight the importance of addressing incorrect risk perceptions of COVID-19, and the continual need to provide psychological interventions to individuals at risk of adverse mental health consequences.

## Data Availability Statement

The datasets generated for this study are not readily available because the conditions of our ethics approval do not permit public archiving of the data supporting this study. Interested researchers seeking access to the anonymized data should contact the corresponding authors and complete a formal data sharing agreement. Access will be granted in accordance with ethical procedures governing the reuse of data. Requests to access the datasets should be directed to MH, pcmhfym@nus.edu.sg or JY, pcmyj@nus.edu.sg.

## Ethics Statement

The studies involving human participants were reviewed and approved by National University of Singapore Institutional Review Board. The patients/participants provided their verbal or written informed consent to participate in this study.

## Author Contributions

MH, RM, and JY were involved in the study design and data collection. MH conducted all data analyses and drafted the manuscript. RM and JY provided input for the manuscript. JY took on a supervisory role in data analyses. All authors contributed to the article and approved the submitted version.

## Conflict of Interest

The authors declare that this study received funding from Research Donations from Kwan Im Thong Hood Cho Temple, Lee Kim Tah Holdings Pte Ltd., and the Hongkong and Shanghai Banking Corporation. The funders were not involved in the study design, collection, analysis, interpretation of data, the writing of this article or the decision to submit it for publication.

## Footnotes

1 The Disease Outbreak Response System Condition is a color-coded framework that highlights the current disease condition in Singapore. Four color statuses (i.e., green, yellow, orange, and red) represent the severity and spread of the disease. The green status represents the lowest alert level, while the red status represents the highest alert level.
