# Supplementary material for: Associations Between Fear of COVID-19, Affective Symptoms and Risk Perception Among Community-Dwelling Older Adults During a COVID-19 Lockdown
Source: Front Psychol. 2021 Mar 23;12:638831. doi: 10.3389/fpsyg.2021.638831 (PMC8021922; doi:10.3389/fpsyg.2021.638831)
Supplement: Supplementary file 1 [file Data_Sheet_1.docx]

**Supplementary Table 1.** Items of the COVID-19 Fear Inventory.

| Item |
| --- |
| 1. To what extent are you afraid of being infected with COVID-19? |
| 1. If you are infected with COVID-19, to what extent are you afraid that you will be severely ill, or die? |
| 1. To what extent are you afraid that your loved ones will be infected with COVID-19? |
| 1. If your loved ones are infected with COVID-19, to what extent are you afraid that they will be severely ill, or die? |
| 1. To what extent are you afraid that COVID-19 is spreading quickly in Singapore? |
| 1. If you are infected with COVID-19, to what extent are you afraid that you will transmit the virus to your loved ones? |
| 1. To what extent has the threat of COVID-19 influenced you to practice social distancing? |
| 1. To what extent has the threat of COVID-19 influenced your use of protective supplies (e.g. hand sanitizer and surgical mask)? |
| 1. To what extent are you worried that protective supplies and other essential items will run out? |
| 1. To what extent do you engage in panic buying (i.e. buying large amounts of protective supplies and other essential items because of the threat of COVID-19)? |
| 1. To what extent are you worried that the threat of COVID-19 could affect your household income? |
| 1. To what extent are you worried that the threat of COVID-19 could affect your personal relationships? |
| 1. To what extent has the threat of COVID-19 influenced you to experience more negative emotions than usual (e.g. fear, worry, panic, etc.)? |

*Note.* Items are rated from 1 (“not at all”) to 5 (“very much”).

**Supplementary Table 2**. Items of the Risk of Infection Questionnaire.

| Without wearing a mask, how likely do you think you will be infected with the COVID-19 virus if you are: |
| --- |
| 1. In a train like this:   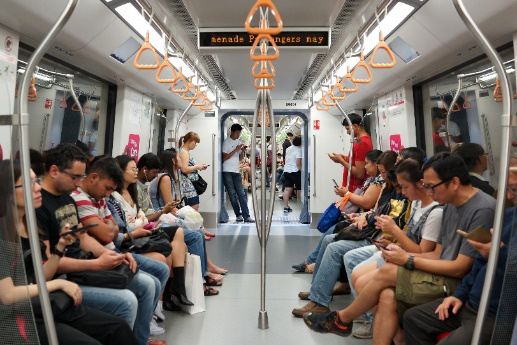 |
| 1. Pressing the lift buttons. |
| 1. In the lift together with 5 other strangers. |
| 1. Sitting on the toilet seat in a public toilet. |
| 1. Eating in a crowded hawker center.   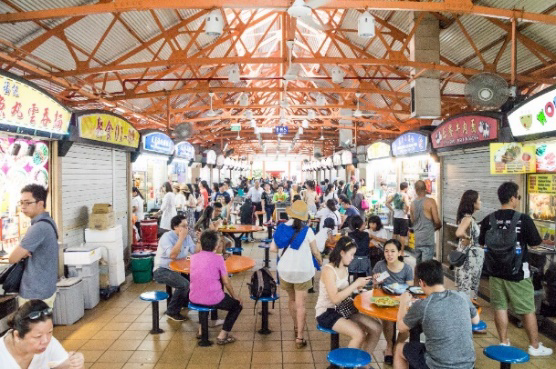 |
| 1. Queuing up in the supermarket.   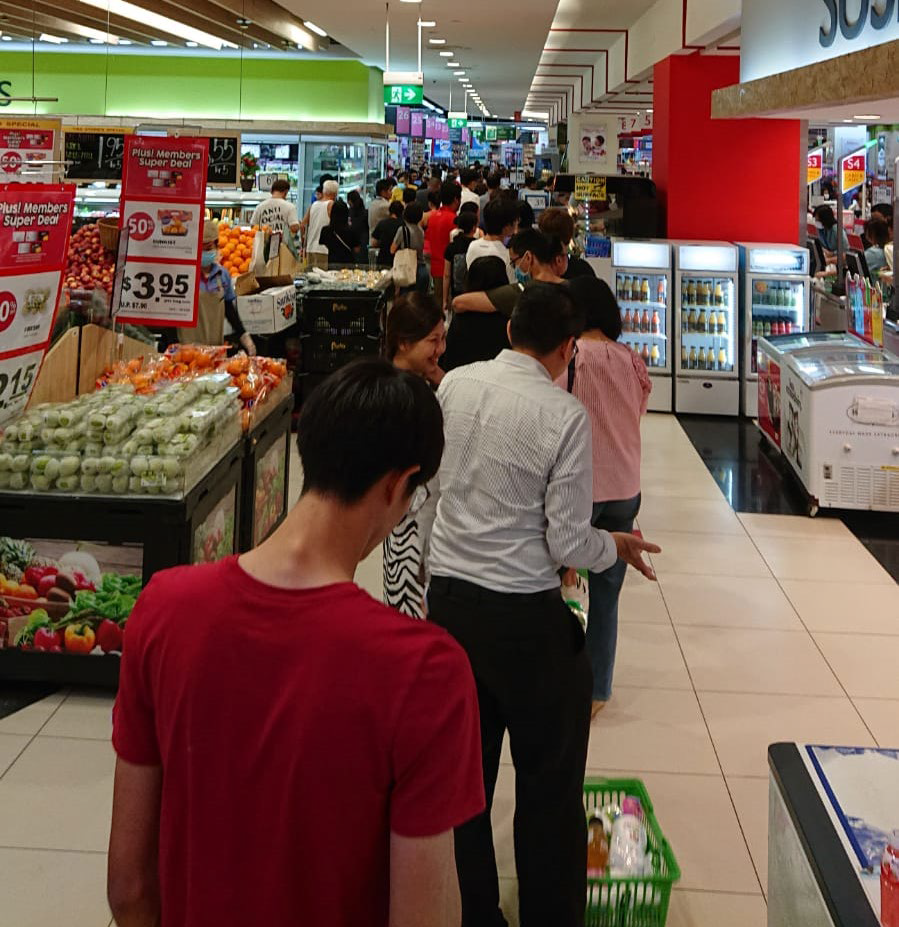 |
| 1. Having 5 friends at your house. |
| 1. Taking a stroll in the park like this:   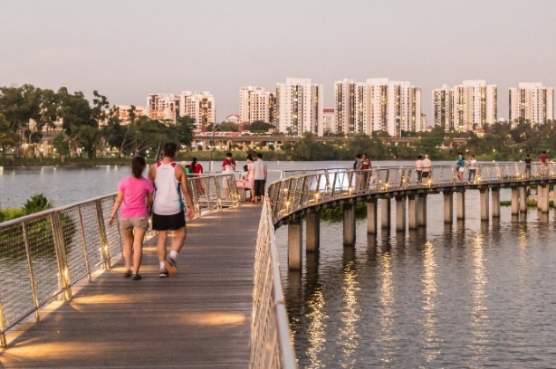 |
| 1. Being around doctors, nurses and other hospital staff. |
| 1. Waiting to see the doctor in a clinic or hospital.   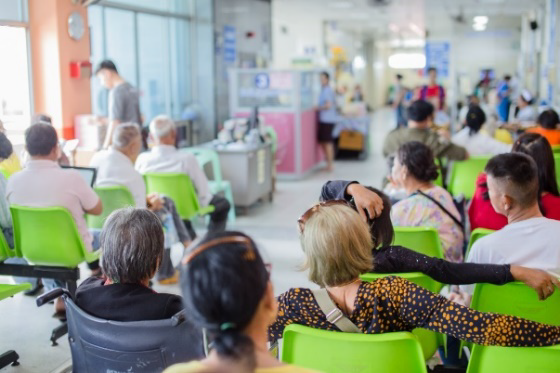 |

*Note.* Items are rated from 1 (“very unlikely”) to 4 (“very likely”).

**Supplementary Table 3**

Fit indices for the CFA models for COVID-19 Fear Inventory and Risk of Infection Questionnaire.

| Model | *χ*^2^ (*df*) | CFI | RMSEA | SRMR | AIC | BIC |
| --- | --- | --- | --- | --- | --- | --- |
| COVID-19 Fear Inventory |  |  |  |  |  |  |
| Model 1 | 552.31 (65) | 0.77 | 0.14 | 0.09 | 14681.25 | 14838.16 |
| Model 2 (removal of items 7, 8, 10, 11) | 54.89 (19) | 0.98 | 0.07 | 0.03 | 10294.81 | 10435.64 |
| Risk of Infection Questionnaire |  |  |  |  |  |  |
| Model 1 | 236.94 (35) | 0.81 | 0.14 | 0.07 | 7776.80 | 7897.44 |
| Model 2 (removal of items 2 and 9) | 61.01 (19) | 0.95 | 0.07 | 0.04 | 6044.06 | 6144.59 |

*Note.* df = degrees of freedom; CFI = Comparative Fit Index; RMSEA = Root Mean Square Error of Approximation; standardized root mean square residual; AIC = Akaike Information Criterion; BIC = Bayesian Information Criterion
